# miR-124-3p Inhibits Microglial Secondary Inflammation After Basal Ganglia Hemorrhage by Targeting TRAF6 and Repressing the Activation of NLRP3 Inflammasome

**DOI:** 10.3389/fneur.2021.653321

**Published:** 2021-08-03

**Authors:** Yudan Fang, Xiaoqin Hong

**Affiliations:** Department of Neurology, Affiliated Hangzhou First People's Hospital, Zhejiang University School of Medicine, Hangzhou, China

**Keywords:** microglia secondary inflammation, intracerebral hemorrhage in basal ganglia, miR-124-3p, TRAF6, NLRP3 inflammasome, inflammatory factors

## Abstract

**Objectives:** Intracerebral hemorrhage (ICH) represents a serious central nervous system emergency with high morbidity and mortality, and the basal ganglia is the most commonly affected brain region. Differentially expressed microRNAs (miRs) have recently been highlighted to serve as potential diagnostic biomarkers and therapeutic targets for ICH. This study investigated the mechanism of miR-124-3p in microglial secondary inflammation after ICH.

**Methods:** In this study, 48 patients with primary basal ganglia ICH and 48 healthy volunteers were selected and venous blood was collected from all patients on the second morning of admission (within 24 h of stroke onset). The expression of miR-124-3p in serum was detected by RT-qPCR. Three months after ICH, the patients were assessed by modified Rankin Scale (mRS), and the correlation between miR-124-3p expression and mRS score was analyzed by Pearson. The inflammatory response of microglia was induced by lipopolysaccharide (LPS) to establish the cell model of microglial inflammation. miR-124-3p expression patterns were detected in the serum of ICH patients and healthy volunteers, normal microglia, and LPS-induced microglia. The miR-124-3p mimic was transfected into LPS-induced microglia, followed by measurement of the inflammatory factors, apoptosis rate, and cell viability. The target gene of miR-124-3p was predicted and verified. The expression patterns of tumor necrosis factor receptor-associated factor 6 (TRAF6) were detected. pcDNA3.1 and pcDNA3.1-TRAF6 were transfected into LPS-induced HMC3 cells, and nucleotide-binding oligomerization domain-like receptor (NLR) pyrin domain-containing 3 (NLRP3) expression patterns were determined. Lastly, the effects of TRAF6 overexpression on apoptosis, cell viability, and inflammation in HMC3 cells were measured.

**Results:** miR-124-3p was downregulated in the serum of basal ganglia ICH patients and LPS-induced microglia, and miR-124-3p expression was negatively correlated with mRS. Overexpression of miR-124-3p reduced the inflammatory factors and apoptosis rate and promoted cell activity in LPS-induced microglia. miR-124-3p was found to target TRAF6. Overexpression of TRAF6 enhanced the expression of NLRP3 inflammasome, inflammatory factors and apoptosis rate, and reduced cell viability.

**Conclusion:** Our findings indicate that miR-124-3p repressed the activation of NLRP3 inflammasome by targeting TRAF6, thus inhibiting microglial secondary inflammation after ICH in basal ganglia.

## Introduction

Intracerebral hemorrhage (ICH) is defined as a primary, spontaneous, and non-traumatic hemorrhage of the brain parenchyma, representing the most fatal type of stroke, with up to 50 mortality of patients in hospital and most survivors manifesting with severe disability (1). ICH is common in the cerebral lobe, basal ganglia, thalamus, brainstem, and cerebellum, which is credited to the rupture of minor penetrating arteries from basilar arteries, or anterior, middle, and posterior cerebral arteries (2). Principally, ICH is manifested as a sudden attack of focal neurological deficit, spanning from minutes to hours, with the manifestation of other associated symptoms including epilepsy, vomiting, headache, and decreased consciousness (3). ICH is an intricate event consequent from the interaction and overlapping of various risk factors. Hypertension persists as the most crucial risk factor, followed by cerebral amyloid angiopathy; however, factors such as smoking, warfarin anticoagulation, excessive drinking, and cocaine cannot be neglected as they can increase the risk (4). Despite the high rates of incidence and mortality, research necessitates for the development of definite interventions to improve the clinical prognosis after ICH (5). The predilection site of ICH is in the area of the basal ganglia (6). Hence, further elucidating the underlying mechanism of ICH in the basal ganglia and developing novel therapeutic targets for ICH patients are urgent for improving the patients' quality of life and reducing the burden of social medicare.

Inflammation essentially manifests immediately after ICH and persists for several days, which may exacerbate the progress of secondary brain injury induced by ICH (7). Microglia are vital immune cells in the central nervous system and hence are implicated in brain development, neural environment maintenance, and response to injury and repair (8). Notably, microglia are alleged as the earliest non-neuronal cells to respond to acute brain injuries, including ICH (8). The activation of microglia corresponds to the secretion of pro-inflammatory cytokines, reactive oxygen species, and matrix metalloproteinases, thus resulting in blood–brain barrier damage and neuronal injury after ICH (9). Currently, the detailed mechanisms underlying the microglial secondary inflammation after ICH remain largely uncertain.

microRNAs (miRs) have emerged as small endogenous non-coding RNAs (18–22 nucleotides long) and implicated in the modulation of gene expression at the post-transcriptional level (10). Existing evidence has acknowledged the differentially expressed miRs and their possible mRNA targets and related pathways as potential diagnostic biomarkers and therapeutic targets for the treatment of ICH (11). miR-124 is highly expressed in the brain of human and rodents, due to its involvement in M2 polarization of microglia and as a protective agent against cerebral inflammation after ICH (12). miR-124-3p, as one of the subtypes of miR-124, biologically functions similarly to the miR-124 family (13). An increased miR-124-3p expression in microglial exosomes after traumatic brain injury represses the inflammatory responses of neurons and exacerbates the neurite outgrowth (14). However, whether miR-124-3p can regulate the secondary inflammatory reaction of microglia and play a protective role in ICH has not been identified yet. This study herein investigates the specific mechanism of miR-124-3p in microglial secondary inflammation after basal ganglia hemorrhage, which shall provide novel insights for the management of ICH.

## Materials and Methods

### Ethics Statement

This study was conducted with approval of the Ethics Committee of the Affiliated Hangzhou First People's Hospital, Zhejiang University School of Medicine. All participants provided written informed consent prior to enrollment.

### Collection of ICH Samples

A total of 59 patients diagnosed with ICH in the Affiliated Hangzhou First People's Hospital, Zhejiang University School of Medicine, from July 2015 to July 2019 were enrolled in this study. To evaluate the relationship between miR-124-3p expression and neurological deficits, we isolated venous blood samples from 48 patients with primary basal ganglia hemorrhage and 48 healthy volunteers visiting the Affiliated Hangzhou First People's Hospital, Zhejiang University School of Medicine, for a conventional physical examination. The inclusion criteria were as follows: (a) identification of patients with primary ICH within 24 h of the disease onset and collection of blood from the cubital fossa vein before the use of any agents; (b) computed tomography-verified basal ganglia hemorrhage; and (c) diagnosis in accordance with the criteria of the European Stroke Initiative (15). The exclusion criteria were as follows: (a) age <18 or >80 years; (b) a history of surgery in the last 6 months; (c) coma or death within 48 h after admission; (d) hematoma resultant of trauma, drug abuse, brain tumor, vascular malformation, anticoagulant therapy, or coagulation dysfunction; (e) grave inflammatory diseases (such as infectious diseases, systemic lupus erythematosus, or rheumatoid arthritis); (f) any hospital-acquired infection; (g) acute and chronic hepatopathy; and (h) diabetes. In total, 48 patients were included in the current study with the exclusion of 11 patients, among which four patients died and seven patients were lost during the follow-up period. Meanwhile, 48 healthy volunteers who visited the Affiliated Hangzhou First People's Hospital, Zhejiang University School of Medicine, for conventional physical examination were selected as the control group. The general clinical features of patients with ICH and healthy volunteers are shown in Table 1. The fasting venous blood was collected from all patients in the morning of the 2nd day after admission, centrifuged at 200 g for 15 min, and allowed to stand, and then the supernatant was obtained and preserved at −80°C. Referring to the previous study (16), the patients were assessed with the modified Rankin Scale (mRS) 3 months after ICH.

**Table 1:** Clinical features of ICH patients and healthy controls.

	**HC**	**ICH**	***p***
Age, y	48 (9.8)	48 (10.12)	0.68
Male, %	55.2	60.1	0.88
History of vascular risk factors, %	No	No	
Blood pressure	88.7	94.2	0.42
Drinking	35.6	37.2	0.68
Smoking habits (current)	33.4	33.1	0.62
Diabetes	No	No	
**Laboratory parameters**			
White blood cell count, ×10^9^/L	7.34 (1.82)	7.62 (1.47)	
Serum glucose, mmoL/L	6.14 (2.14)	6.53 (1.79)	0.691
platelet count, ×10^9^/L	199 (50)	217 (36)	0.755
Obvious inflammation occurred within 6 months	No	No	
Acute myocardial infarction	No	No	
Acute or chronic liver injury	No	No	

### Cell Transfection and Establishment of Microglial Inflammation Model

HMC3 cells (American Type Culture Collection, Manassas, VA, USA), miR-124-3p mimic, pcDNA3.1-tumor necrosis factor receptor-associated factor 6 (TRAF6), si-TRAF6, and corresponding controls were designed and synthesized by GenePharma (Shanghai, China). HMC3 cells were cultured in Dulbecco's modified Eagle's medium (DMEM; A4192101, GIBCO, Grand Island, NY, USA) containing a combination of 10% fetal bovine serum (FBS; 16000044, GIBCO), 100 U/ml penicillin, and 100 g/ml streptomycin at 37°C. The cell suspension (about 1 × 10^4^ cells) was seeded into 24-well plates for incubation at 37°C with 5% CO_2_. HMC3 cells in the logarithmic phase were seeded into the six-well plates (3 × 10^5^/ml). The cells were transfected using Lipofectamine^®^ 2000 upon growing adherent to the surface. After 48 h, the cells were isolated for subsequent experiments. The lipopolysaccharide (LPS) powder was dissolved in mother liquor at a concentration of 1 mg/ml with phosphate-buffered saline (PBS), after which the cells were seeded into 12-well plates (20 × 10^4^ cells/well) containing LPS (the final solubility was 1μg/ml) (17) for incubation at 37°C with 5% CO_2_. After 24 h, the cells were isolated for subsequent experimentation.

The cells were assigned as follows: the normal group (untreated HMC3 cells), the LPS group (LPS-treated HMC3 cells), the LPS + mimic negative control (NC) group (LPS-treated HMC3 cells were transfected with mimic NC), the LPS + miR-124-3p mimic group (LPS-treated HMC3 cells were transfected with miR-124-3p mimic), the LPS + NC group (LPS-treated HMC3 cells were transfected with empty vector), the LPS + p-TRAF6 group (LPS-treated HMC3 cells were transfected with pcDNA3.1-TRAF6), the LPS + si-NC group (LPS-treated HMC3 cells were transfected with si-NC), the LPS + si-TRAF6 group (LPS-treated HMC3 cells were transfected with si-TRAF6), and the LPS + p-TRAF6 + miR-124-3p mimic group (LPS-treated HMC3 cells were transfected with pcDNA3.1-TRAF6 and miR-124-3p mimic), respectively.

### Enzyme-Linked Immunosorbent Assay (ELISA)

The levels of interleukin (IL)-1β, IL-6, and tumor necrosis factor (TNF)-α in the cell supernatant or brain tissues were analyzed using the ELISA kit (R&D Systems, Minneapolis, MN, USA). After 24 h of stimulation, the cell supernatant was collected and preserved at −70°C.

### Reverse Transcription Quantitative Polymerase Chain Reaction (RT-qPCR)

The total RNA content was extracted from the clinical sample tissues or cultured cells using the TRIzol reagent (Invitrogen Inc., Carlsbad, CA, USA) and identified by a combination of UV analysis and formaldehyde denaturing gel electrophoresis. RT-qPCR was performed on ABI 7500 (ABI Inc., Foster City, CA, USA), with U6 and β-actin serving as an internal reference. The relative expression of genes was calculated based on the 2-^ΔΔCT^ method. The formula was as follows: ΔΔCT = [Ct (target gene) – Ct (reference gene)] experimental group - [Ct (target gene) – Ct (reference gene)] control group. The primers are shown in Table 2.

**Table 2:** Primer sequence for RT-qPCR.

**Name of primer**	**Sequences**
miR-124-3p forward	5′-GCCTAAGGCACGCGGTG-3′
miR-124-3p reverse	5′-GTCGTATCCAGTGCAGGGTCCGAGGTATTCGCACTGGA TACGACGGCATT-3′
TRAF6 forward	5′-CAGTGGTCGTATCGTGCTTA-3′
TRAF6 reverse	5′-CCTTA TGGTTTCTTGGAGTC-3′
U6 forward	5′-CTCGCTTCGGCAGCACA-3′
U6 reverse	5′-AACGCTTCACGAATTTGCGT-3′
β-actin forward	5′-GTTGCGTTACACCCTTTCTTG-3′
β-actin reverse	5′-GACTGCTGTCACCTTCACCGT-3′

### Western Blotting

After 48 h of transfection, the protein content was extracted from the LPS-induced microglia in radio-immunoprecipitation assay buffer. The protein concentration of the supernatant was measured using the bicinchoninic acid assay. The protein (50 g) was separated on 5–15% sodium dodecyl sulfate-polyacrylamide gel electrophoresis and transferred onto polyvinylidene fluoride membranes. Membrane blockade was conducted using 5% skim milk–Tris-buffered saline Tween (TBST; 10mM Tris–HCl, ph 7.5, 150mM NaCl and 0.1% Tween-20) for 1 h and incubated with the corresponding primary antibodies anti-human TRAF6 polyclonal antibody (dilution ratio of 1:500, ab181622, Abcam Inc., Cambridge, MA, USA) and rabbit anti-human β-actin monoclonal antibody (dilution ratio of 1:1,000, ab8227, Abcam) at 4°C overnight. The membranes were rinsed with TBST three times (5 min/time) and incubated with the corresponding goat anti-rabbit immunoglobulin G (IgG) (dilution ratio of 1:2,000, A6154, Sigma-Aldrich, Merck KGaA, Darmstadt, Germany) at 37°C for 1 h. Next, the membranes were developed and visualized using the enhanced chemiluminescence reagent (Thermo Fisher Scientific Inc., Waltham, MA, USA). The gray value of each band was quantified using PDQuest 8.0 (Bio-Rad, Hercules, CA, USA). The relative protein level of TRAF6 was estimated as the gray value ratio of TRAF6/β-actin.

### Dual-Luciferase Reporter Gene Assay

The 3′-UTR fragment of TRAF6-wild type (WT) complementary to miR-124-3p was synthesized. Next, the TRAF6-WT and TRAF6-mutant type (MUT) containing the binding sequence and mutation sequence of miR-124-3p were cloned into the pmiR-GLO luciferase vector. The cultured microglia were thoroughly seeded into six-well plates (3 × 10^5^/ml). The constructed luciferase plasmids were co-transfected with mimic NC or miR-143-3p mimic into the microglia. The cells were isolated 48 h later, and the luciferase activity was analyzed using the dual-luciferase reporter gene assay kit. The values were measured the wavelength of at 560 nm on the microplate reader (BioTek Inc., Norcross, GA, USA).

### Flow Cytometry

The LPS-induced microglia were cultured using Annexin V-FITC and propidium iodide in conditions devoid of light for 15 min. The cells were filtered, after which the cell apoptosis was evaluated by flow cytometry (BD Biosciences, San Jose, CA, USA). The cells were counted using the CellQuest Software (BD Biosciences), and the data were analyzed using the Mac Quit Software.

### 3-(4,5-Dimethylthiazol-2-yl)-2,5-Diphenyltetrazolium Bromide (MTT) Assay

After 48 h of transfection, the microglia were cultured using 100 μl MTT solution (0.5 mg/ml/well) at 37°C for 4 h. Each well was treated with 100 μl 20% sodium dodecyl sulfate (auxiliary solvent, 50% dimethylformamide) at 37°C for 24 h. The optical density was measured at the wavelength of 570 nm on the microplate reader.

### Statistical Analysis

Data analysis was conducted using the SPSS 21.0 (IBM Corp., Armonk, NY, USA). The Kolmogorov–Smirnov method was adopted to evaluate whether the data were in normal distribution. Data are expressed as mean ± standard deviation. The unpaired *t*-test was adopted for comparison between two groups. One-way analysis of variance (ANOVA) was employed for comparisons among multiple groups, followed by Tukey's multiple-comparison test. Pearson was used to analyze the correlation between the serum miR-124-3p level and the mRS score 3 months after ICHe. The *p*-value was estimated from a two-tailed test, and a value of *p* < 0.05 was of statistical significance.

## Results

### miR-124-3p Was Downregulated in Microglia After ICH

**Figure 1:**
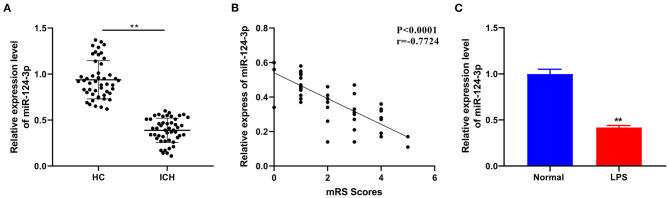
miR-124-3p was downregulated in microglia after ICH. **(A)** The expression of miR-124-3p in the serum of patients with primary ICH in the basal ganglia was detected using RT-qPCR, *N* = 48; **(B)** Pearson was used to analyze the correlation between the expression of miR-124-3p and the modified Rankin Scale (mRS) score 3 months after ICH; **(C)** The expression of miR-124-3p in LPS-induced microglia and normal microglia was detected using RT-qPCR. The cell experiment was repeated three times independently. Data are expressed as mean ± standard deviation. Data in panels A/C were analyzed using *t*-test, ^**^p < 0.05.

As one of the most extensively present miRs in the brain, miR-124-3p is essential for the development of the nervous system and is regarded as a vital regulator of microglial function (18). To determine the role of miR-124-3p in ICH, we selected 48 patients with primary basal ganglia ICH and 48 healthy volunteers and venous blood was collected from all patients on the second morning of admission. The miR-124-3p expression pattern in ICH patients and healthy volunteers was detected by RT-qPCR, and the results revealed that the miR-124-3p expression pattern was considerably lower in the serum of ICH patients relative to the healthy volunteers (*p* < 0.05, Figure 1A). In addition, we evaluated the mRS score 3 months after ICH and analyzed the correlation between miR-124-3p expression and mRS score by Pearson. The results revealed that the miR-124-3p expression pattern was negatively correlated with the mRS score (*r* = −0.7724, Figure 1B). Human microglial HMC3 cells were induced by LPS to establish an *in vitro* cell model of microglial inflammation. The miR-124-3p expression pattern in LPS-treated cells was notably lower compared to the control cells (*p* < 0.05, Figure 1C). Altogether, we speculated an association between miR-124-3p with microglial injury in ICH.

### Overexpression of miR-124-3p Inhibited LPS-Induced Inflammatory Response in Microglia

**Figure 2:**
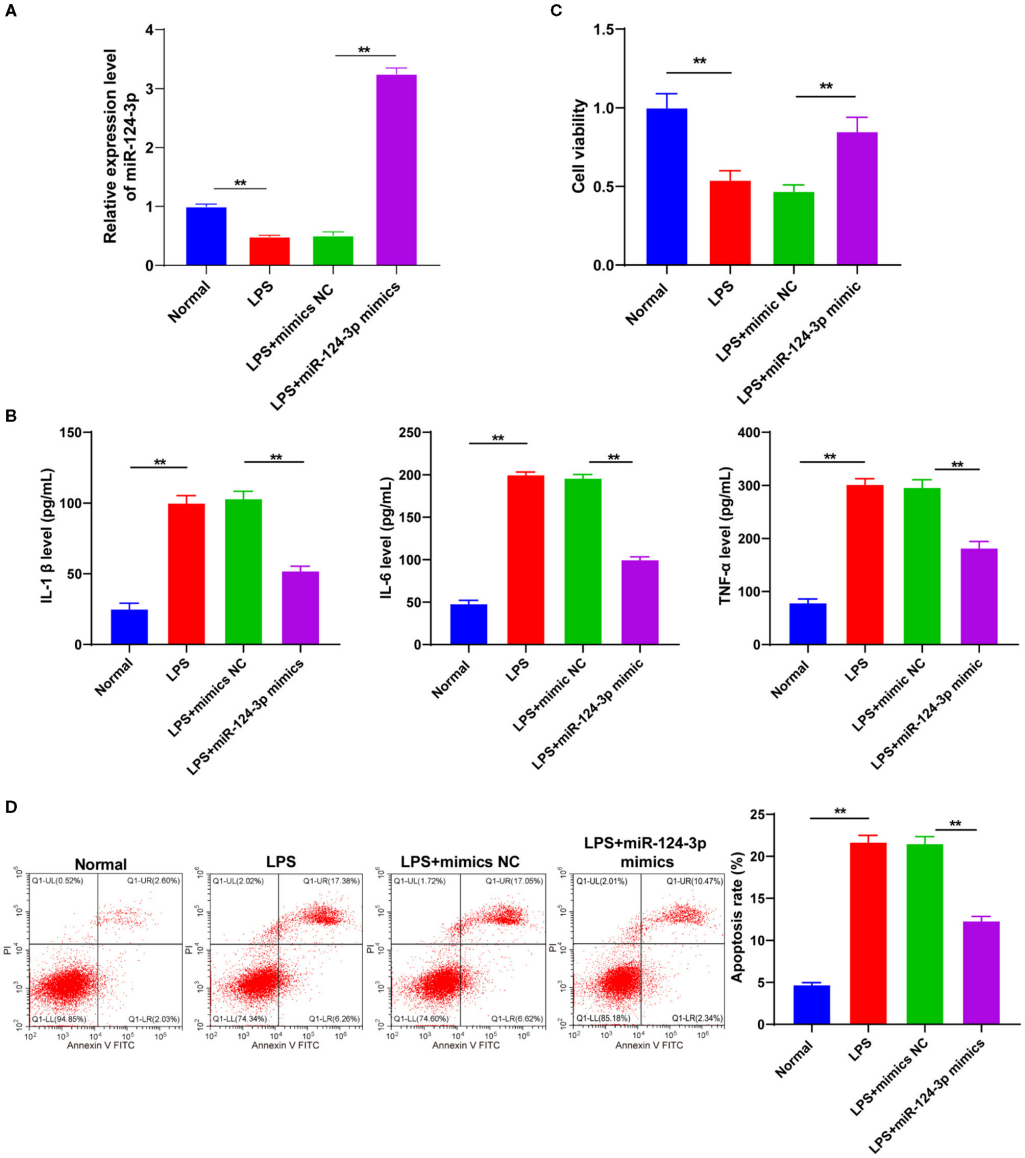
Overexpression of miR-124-3p inhibited LPS-induced inflammatory response and apoptosis in microglia. LPS-induced microglia were transfected with miR-124-3p mimic. **(A)** Transfection efficiency of miR-124-3p was confirmed using RT-qPCR; **(B)** Inflammatory factors IL-1β, IL-6, and TNF-α in microglia were detected using ELISA; **(C)** Cell viability was detected using MTT; **(D)** Cell apoptosis was measured using flow cytometry. The cell experiment was repeated three times independently. Data are expressed as mean ± standard deviation and analyzed using two-way ANOVA, followed by Tukey's multiple-comparison test, ^**^p < 0.05.

To identify the role of miR-124-3p in the microglial secondary inflammation after ICH, we transfected miR-124-3p mimic into the LPS-induced cells. RT-qPCR showed that the miR-124-3p expression pattern in cells of the LPS + miR-124-3p mimic group was notably higher compared to the cells in the control group and the LPS + mimic NC group (*p* < 0.05, Figure 2A). The levels of IL-1β, IL-6, and TNF-α were detected using ELISA, and the results revealed that the levels of inflammatory factors were considerably reduced after miR-124-3p mimic transfection (*p* < 0.05, Figure 2B). MTT analysis revealed that the cell viability of miR-124-3p mimic-treated cells was higher than that of control cells 48 h after transfection (*p* < 0.05, Figure 2C). Flow cytometry showed a significantly reduced apoptosis rate of cells transfected with the miR-124-3p mimic (*p* < 0.05, Figure 2D). These results suggested that miR-124-3p could inhibit inflammation and apoptosis *in vitro*.

### miR-124-3p Targeted TRAF6

**Figure 3:**
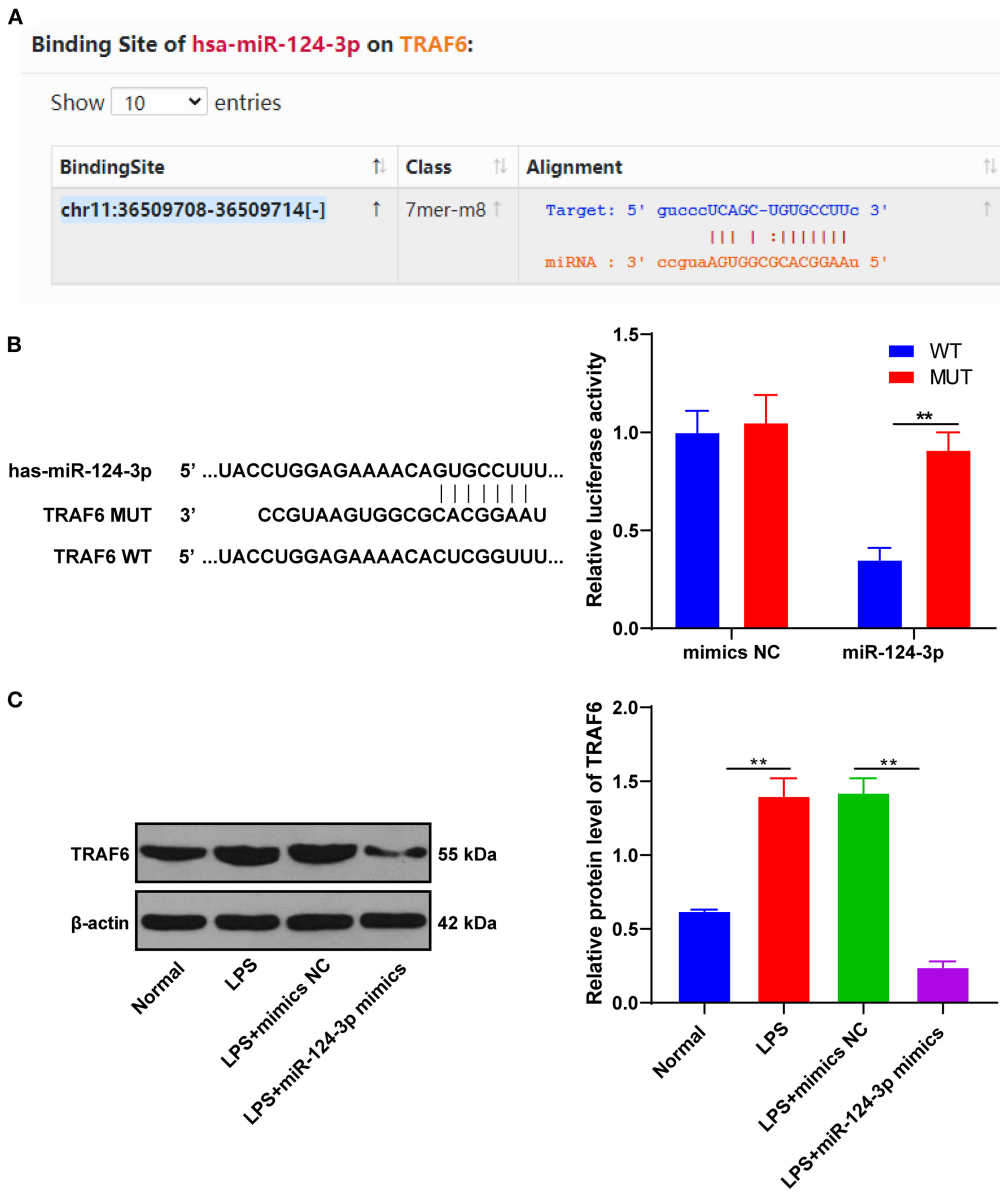
miR-124-3p-targeted TRAF6. **(A)** The binding site between miR-124-3p and TRAF6 was predicted by Starbase; **(B)** The relative luciferase activity of miR-124-3p mimic group and mimic NC group in dual-luciferase reporter gene assay; **(C)** The relative expression of TRAF6 was detected using Western blotting. The cell experiments were repeated three times. Data are expressed as mean ± standard deviation and analyzed using one-way ANOVA, followed by Tukey's multiple-comparison test, ^**^p < 0.05.

To understand the potential mechanism of miR-124-3p in ICH, our prediction through the Starbase identified TRAF6 as the target gene of miR-124-3p (Figure 3A). The binding relationship between miR-124-3p and TRAF6 was verified using dual-luciferase reporter gene assay. The TRAF6 3′-UTR (WT) and TRAF6 3′-UTR (MUT) were constructed and transfected into microglia in combination with the miR-124-3p mimic and mimic NC. The luciferase activity of TRAF6 3′-UTR (WT) in the miR-124-3p mimic group was markedly lower than that in the mimic NC group (*p* < 0.05, Figure 3B). Essentially, miR-124-3p might act directly on the predicted target site of TRAF6. After transfection of miR-124-3p mimic into LPS-induced microglia, the expression pattern of TRAF6 and the regulation of miR-124-3p on the TRAF6 expression pattern were analyzed using Western blotting. The protein level of TRAF6 in the microglia of the miR-124-3p mimic group was remarkably weaker than that in the control group and the mimic NC group (*p* < 0.05, Figure 3C), thus indicating that overexpression of miR-124-3p inhibited the TRAF6 protein level. Briefly, miR-124-3p inhibited the TRAF6 protein level.

### miR-124-3p Reduced Inflammatory Response by Targeting TRAF6

**Figure 4:**
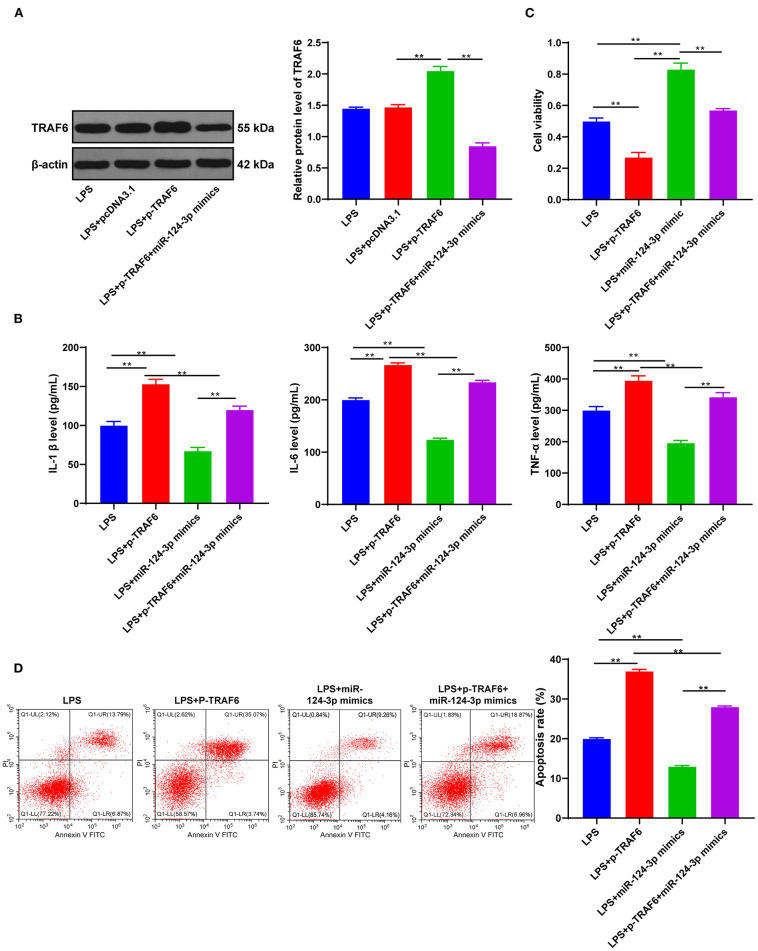
miR-124-3p reduced inflammatory response by targeting TRAF6. **(A)** TRAF6 expression in untreated HMC3 cells, pcDNA3.1- or p-TRAF6-treated HMC3 cells was detected using Western blotting; **(B)** Inflammatory factors IL-1β, IL-6, and TNF-α in p-TRAF6- or miR-124-3p mimic-transfected HMC3 cells induced by LPS were detected using ELISA; **(C)** Cell viability was detected using MTT; **(D)** Cell apoptosis was measured using flow cytometry. The cell experiment was repeated three times independently. Data are expressed as mean ± standard deviation and analyzed using one-way ANOVA, followed by Tukey's multiple-comparison test, ^**^p < 0.05.

To investigate whether TRAF6 was involved in the effect of miR-124-3p overexpression on microglial apoptosis and inflammatory response after ICH, we transfected overexpression empty vector pcDNA3.1 or pcDNA3.1-TRAF6 into LPS-induced HMC3 cells. Subsequent results of Western blotting showed that TRAF6 expression was promoted in HMC3 cells transfected with pcDNA3.1-TRAF6 (*p* < 0.05, Figure 4A). Meanwhile, it was found that overexpression of TRAF6 significantly reversed the downregulation of inflammatory factors induced by miR-124-3p mimic (*p* < 0.05, Figure 4B). Also, overexpression of TRAF6 inhibited the cell viability, while further miR-124-3p mimic was found to reverse the cell viability, yet the cell viability was still lower than that of the control group (*p* < 0.05, Figure 4C). Lastly, overexpression of TRAF6 not only enhanced cell apoptosis but also blocked the protective effect of miR-124-3p mimic on apoptosis (*p* < 0.05, Figure 4D). Briefly, miR-124-3p reduced the inflammatory response by targeting TRAF6.

### TRAF6 Promoted Microglial Secondary Inflammation by Mediating the NLRP3 Inflammatory Pathway

**Figure 5:**
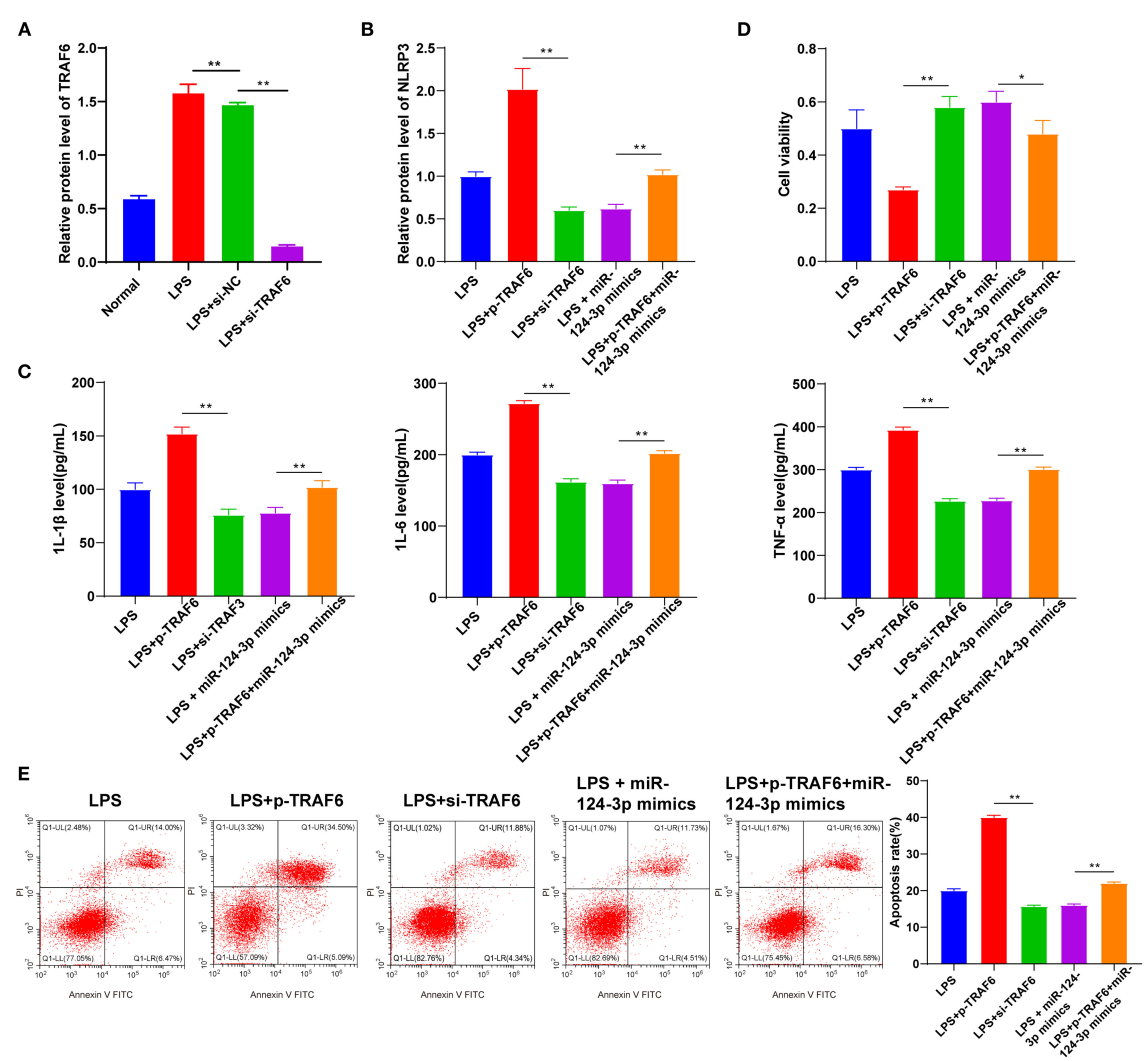
TRAF6 promoted microglial secondary inflammation by mediating the NLRP3 inflammatory pathway. LPS-induced HMC3 cells were transfected with pcDNA3.1, p-TRAF6, si-NC, si-TRAF6, and miR-124-3p mimic. **(A)** Transfection efficiency of TRAF6 was confirmed using RT-qPCR; **(B)** The relative expression of NLRP3 was detected using RT-qPCR; **(C)** The levels of inflammatory factors were detected using ELISA; **(D)** Cell viability was detected using MTT assay; **(E)** Cell apoptosis was measured using flow cytometry. The cell experiment was repeated three times independently. Data are expressed as mean ± standard deviation and analyzed using one-way ANOVA, followed by Tukey's multiple-comparison test, ^*^p < 0.05, ^**^p < 0.01.

TRAF6 is known as an intracellular scaffold protein, which directly or indirectly participates in the signal transduction pathway of the TNF receptor family and the IL-1R and toll-like receptor (TLR) family (19). TRAF6 was further highlighted to initiate downstream signal transduction when binding to activated receptor complexes, especially the activation of the NLRP3 inflammasome (20). However, it is not clear whether TRAF6 regulates the secondary inflammation of microglia after ICH by activating the NLRP3 inflammasome. We transfected LPS-induced microglia with pcDNA3.1-TRAF6 or si-TRAF6 to elucidate the mechanism of TRAF6 in the microglial secondary inflammation. The transfection efficiency was confirmed by RT-qPCR (*p* < 0.05, Figure 5A), and NLRP3 expression was found to be downregulated after inhibition of TRAF6 while being upregulated after overexpression of TRAF6; overexpression of TRAF6 also upregulated inflammatory factors in cells, while inhibition of TRAF6 brought about the opposite effects (*p* < 0.05, Figures 5B,C). In addition, cell viability was noted to be reduced after overexpression of TRAF6 and promoted after inhibition of TRAF6 (*p* < 0.05, Figure 5D). The results further demonstrated that the proportion of apoptotic cells in the p-TRAF6 group was higher than that in the control group, while the proportion of apoptotic cells in the si-TRAF6 group was slightly lower than that in the control group (*p* < 0.05, Figure 5E). Moreover, overexpression of miR-124-3p significantly decreased the expression of NLRP3, downregulated the expressions of inflammatory factors, enhanced cell viability, and reduced apoptosis (*p* < 0.05, Figures 5B–E). On the other hand, overexpression of TRAF6 significantly alleviated the aforementioned effects of miR-124-3p overexpression on microglia. Compared with the LPS + miR-124-3p mimic group, the LPS + p-TRAF6 + miR-124-3p mimic presented with upregulated expressions of NLRP3 and inflammatory factors, reduced cell viability, and increased apoptosis (*p* < 0.05, Figures 5B–E). Taken together, miR-124-3p targeted TRAF6 to regulate the NLRP3 expression, thereby modulating the secondary inflammation of microglia after ICH.

## Discussion

ICH is a life-threatening emergency with poor prognosis and limited treatment protocols (21). The critical role of microglia-mediated inflammation in the manifestation and development of brain injury after ICH has been identified by a team of researchers (22). miR-124 can facilitate the polarization of activated microglia and stimulate migration of macrophages to the anti-inflammatory M2 phenotype, thereby functioning as a protective barrier for neurons after brain injury (23). Our study elucidated the mechanism of miR-124-3p/TRAF6 in the regulation of microglial secondary inflammatory response after basal ganglia hemorrhage.

The brain-specific miR-124 serves as a potential biomarker for the early diagnosis of ICH (24). To identify the role of miR-124-3p in ICH, we detected the miR-124-3p expression in the serum of ICH patients and LPS-treated microglia. The results presented with a notably downregulated miR-124-3p expression in the serum of ICH patients and LPS-treated microglia. Additionally, a negative correlation has been identified between miR-124-3p expression and the mRS score in ICH patients. He et al. suggested that an increase in miR-124-3p expression is associated with the prognosis of patients with acute ischemic stroke receiving thrombolysis (25). Our initial findings elicited that the downregulated miR-124-3p expression might be associated with microglial injury after ICH. Neuroinflammation is a vital factor for analysis of nerve injury caused by ICH; the manifestation pathological or physiological injury by the brain initiates activation of inflammatory cells (26). Activated microglia can principally be categorized into two main subtypes: classical activated M1 phenotype and the alternative activated M2 phenotype. The M1 phenotype elicits pro-inflammatory activity with the release of pro-inflammatory factors such as IL-6 and TNF-α, while the M2 phenotype is characteristic of anti-inflammatory activity induced by the release of several anti-inflammatory factors such as IL-10 and TGF-β (27, 28). LPS induces activation of M1 microglia (28), facilitates the apoptosis of microglia, and decreases the cell survival rate (29). Our results demonstrated that overexpression of miR-124-3p could alleviate the damage of LPS to microglia. Moreover, overexpression of miR-124-3p improved the cell survival, inhibited the pro-inflammatory activity of microglia, and induced anti-inflammatory activity. Also, our results elicited the ability of an overexpression of miR-124-3p to significantly reduce LPS-induced inflammation. An existing study has exhibited that increasing the miR-124-3p expression in microglia can facilitate the anti-inflammatory M2 polarization to consequently suppress the inflammatory response of neurons in mice with traumatic brain injury (14). Next, we transfected the miR-124-3p mimic into LPS-induced microglia, where the results indicated the ability of an overexpression of miR-124-3p to markedly reduce the levels of inflammatory factors and apoptosis rate and facilitate cell activity. Consistently, miR-124-3p mimic can repress the levels of inflammation cytokines in rats induced by chronic sciatic nerve injury (30). The miR-124 inhibitors are capable of improving neuronal apoptosis via the Bcl-2/Bcl-xl pathway in ICH (12). Briefly, miR-124-3p inhibited the inflammatory response and cell apoptosis after basal ganglia hemorrhage *in vitro*.

Thereafter, we aimed at identifying the target genes of miR-124-3p in the regulation of microglial secondary inflammation after ICH. Essentially, the miR-124-3p/TRAF6 axis can evidently regulate the inflammatory response and apoptosis induced by ischemia–reperfusion injury in human cardiac myocytes (31). TRAF is a vital binding protein of the TNF and TLR superfamily, with notable involvement in innate immunity and acquired immunity (32). Intriguingly, TRAF6 is implicated in the manifestation of hemorrhagic stroke corresponding to its E3 ubiquitin ligase activity (32). In the current study, the targeting relationship between miR-124-3p and TRAF6 was validated using dual-luciferase reporter gene assay. Upon transfection of pcDNA3.1 and pcDNA3.1-TRAF6 into the LPS-induced HMC3 cells, the effects of TRAF6 overexpression on the apoptosis, cell viability, and inflammatory response of HMC3 cells were thoroughly evaluated. TRAF6 overexpression can improve the levels of inflammatory factors and apoptosis rate and inhibit cell activity. Previous research has documented the protective effects of TRAF6 inhibition on ICH by restraining the degree of inflammation and oxidative stress (33). Yang et al. have also revealed that luteolin binds to TRAF6 and represses the ubiquitination of TRAF6 to function as a neuroprotective marker in ICH (34). Conjointly, miR-124-3p reduced LPS-induced inflammation in HMC3 cells by targeting TRAF6 expression.

TRAF6 has been documented to critically function in the TLR/IL-1R-initiated activation of NLRP3 inflammasome (20). As a pivotal regulator of the innate immune system, NLRP3 is activated after ICH (35), which can boost the inflammatory response by the release of IL-1β and facilitate neutrophil infiltration, thus radically exacerbating brain edema (36). The speculation regarding whether TRAF6 regulated the secondary inflammation of microglia after ICH by activating NLRP3 inflammasome was uncertain. Hence, the HMC3 cells were transfected with p-TRAF6 or si-TRAF6 to determine the mechanism of TRAF6 in secondary inflammation of microglia. Upon inhibition of TRAF6, the NLRP3 expression would decrease; however, upon overexpression of TRAF6, the NLRP3 expression would increase. Song et al. have demonstrated that the ability of repressing the activation of the NLRP3 inflammasome can weaken secondary brain injury and inflammatory reaction and protect the integrity and permeability of the blood–brain barrier after ICH (37). Our results verified that TRAF6 promoted microglial secondary inflammation after ICH by enhancing the NLRP3 inflammasome.

To conclude, miR-124-3p inhibits microglial secondary inflammation after basal ganglia hemorrhage by targeting the TRAF6 expression and repressing the activation of the NLRP3 inflammasome. Our study may speculate the functionality of miR-124-3p as a potential target for ICH patients. Our future researches shall focus on a comprehensive investigation of the extensive regulatory mechanism of the NLRP3 inflammasome, as well as the feasibility and safety of miR-124-3p in the clinical treatment of ICH.

## Data Availability Statement

The original contributions presented in the study are included in the article/supplementary material, further inquiries can be directed to the corresponding author.

## Ethics Statement

The studies involving human participants were reviewed and approved by Affiliated Hangzhou First People's Hospital, Zhejiang University School of Medicine. The patients/participants provided their written informed consent to participate in this study.

## Author Contributions

XH was the guarantor of integrity of the entire study and contributed to the study concepts, study design, and definition of intellectual content. YF contributed to the literature research, clinical studies, experimental studies, data acquisition, data analysis, statistical analysis, manuscript preparation, manuscript editing, and manuscript review. All the authors read and approved the final manuscript.

## Conflict of Interest

The authors declare that the research was conducted in the absence of any commercial or financial relationships that could be construed as a potential conflict of interest.

## Publisher's Note

All claims expressed in this article are solely those of the authors and do not necessarily represent those of their affiliated organizations, or those of the publisher, the editors and the reviewers. Any product that may be evaluated in this article, or claim that may be made by its manufacturer, is not guaranteed or endorsed by the publisher.

## Abbreviations

ICH: intracerebral hemorrhage
miRs: microRNAs
LPS: lipopolysaccharide
mRS: modified Rankin Scale
TRAF6: tumor necrosis factor receptor-associated factor
NLRP3: nucleotide-binding oligomerization domain-like receptor (NLR) pyrin domain-containing 3
DMEM: Dulbecco's modified Eagle's medium
FBS: fetal bovine serum
PBS: phosphate-buffered saline
IL: interleukin
ELISA: enzyme-linked immunosorbent assay
IgG: immunoglobulin G
RT-qPCR: reverse transcription quantitative polymerase chain reaction
TNF: tumor necrosis factor
TLR: toll-like receptor
TBST: Tris buffered saline Tween
ANOVA: analysis of variance.

## References

[1] Morotti A Goldstein JN. Diagnosis and management of acute intracerebral hemorrhage. Emerg Med Clin North Am. (2016) 34:883–99. 10.1016/j.emc.2016.06.010 27741993 PMC5089075

[2] Van Matre ET Sherman DS Kiser TH. Management of intracerebral hemorrhage–use of statins. Vasc Health Risk Manag. (2016) 12:153–61. 10.2147/VHRM.S75399 27143909 PMC4841406

[3] Rymer MM. Hemorrhagic stroke: intracerebral hemorrhage. Mol Med. (2011) 108:50–4. PMC6188453 21462612

[4] Ikram MA Wieberdink RG Koudstaal PJ. International epidemiology of intracerebral hemorrhage. Curr Atheroscler Rep. (2012) 14:300–6. 10.1007/s11883-012-0252-1 22538431 PMC3388250

[5] Garg R Biller J. Recent advances in spontaneous intracerebral hemorrhage. F1000Res. (2019). 8:16357. 10.12688/f1000research.16357.1 30906532 PMC6426087

[6] Li J Xiao L He D Luo Y Sun H. Mechanism of white matter injury and promising therapeutic strategies of MSCs after intracerebral hemorrhage. Front Aging Neurosci. (2021) 13:632054. 10.3389/fnagi.2021.632054 33927608 PMC8078548

[7] Wu H Zhang Z Hu X Zhao R Song Y Ban X. Dynamic changes of inflammatory markers in brain after hemorrhagic stroke in humans: a postmortem study. Brain Res. (2010) 1342:111–7. 10.1016/j.brainres.2010.04.033 20420814 PMC2885522

[8] Li M Li Z Ren H Jin WN Wood K Liu Q. Colony stimulating factor 1 receptor inhibition eliminates microglia and attenuates brain injury after intracerebral hemorrhage. J Cereb Blood Flow Metab. (2017) 37:2383–95. 10.1177/0271678X16666551 27596835 PMC5482387

[9] Taylor RA Chang CF Goods BA Hammond MD Mac Grory B Ai Y. TGF-beta1 modulates microglial phenotype and promotes recovery after intracerebral hemorrhage. J Clin Invest. (2017) 127:280–92. 10.1172/JCI88647 27893460 PMC5199690

[10] Kim VN Nam JW. Genomics of microRNA. Trends Genet. (2006) 22:165–73. 10.1016/j.tig.2006.01.003 16446010

[11] Cheng X Ander BP Jickling GC Zhan X Hull H Sharp FR. MicroRNA and their target mRNAs change expression in whole blood of patients after intracerebral hemorrhage. J Cereb Blood Flow Metab. (2020) 40:775–86. 10.1177/0271678X19839501 30966854 PMC7168793

[12] Yu A Zhang T Duan H Pan Y Zhang X Yang G. MiR-124 contributes to M2 polarization of microglia and confers brain inflammatory protection via the C/EBP-alpha pathway in intracerebral hemorrhage. Immunol Lett. (2017) 182:1–11. 10.1016/j.imlet.2016.12.003 28025043

[13] Kang Q Xiang Y Li D Liang J Zhang X Zhou F. MiR-124-3p attenuates hyperphosphorylation of Tau protein-induced apoptosis via caveolin-1-PI3K/Akt/GSK3beta pathway in N2a/APP695swe cells. Oncotarget. (2017) 8:24314–26. 10.18632/oncotarget.15149 28186985 PMC5421849

[14] Huang S Ge X Yu J Han Z Yin Z Li Y. Increased miR-124-3p in microglial exosomes following traumatic brain injury inhibits neuronal inflammation and contributes to neurite outgrowth via their transfer into neurons. FASEB J. (2018) 32:512–28. 10.1096/fj.201700673r 28935818

[15] European Stroke Initiative Writing C Writing Committee for the EEC Steiner T Kaste M Forsting M Mendelow D. Recommendations for the management of intracranial haemorrhage - part I: spontaneous intracerebral haemorrhage. The European Stroke Initiative Writing Committee and the Writing Committee for the EUSI Executive Committee. Cerebrovasc Dis. (2006) 22:294–316. 10.1159/000094831 16926557

[16] Meng Z Zhao T Zhou K Zhong Q Wang Y Xiong X. A20 ameliorates intracerebral hemorrhage-induced inflammatory injury by regulating TRAF6 polyubiquitination. J Immunol. (2017) 198:820–31. 10.4049/jimmunol.1600334 27986908 PMC5220121

[17] Gao S Cheng QC Hu YG Tan ZZ Chen L Liu SW. LncRNA AK148321 alleviates neuroinflammation in LPS-stimulated BV2 microglial cell through regulating microRNA-1199-5p/HSPA5 axis. Life Sci. (2021) 266:118863. 10.1016/j.lfs.2020.118863 33301806

[18] Svahn AJ Giacomotto J Graeber MB Rinkwitz S Becker TS. miR-124 Contributes to the functional maturity of microglia. Dev Neurobiol. (2016) 76:507–18. 10.1002/dneu.22328 26184457

[19] Chung JY Lu M Yin Q Lin SC Wu H. Molecular basis for the unique specificity of TRAF6. Adv Exp Med Biol. (2007) 597:122–30. 10.1007/978-0-387-70630-6_10 17633022

[20] Xing Y Yao X Li H Xue G Guo Q Yang G. Cutting edge: TRAF6 mediates TLR/IL-1R signaling-induced nontranscriptional priming of the NLRP3 inflammasome. J Immunol. (2017) 199:1561–6. 10.4049/jimmunol.1700175 28739881

[21] Cordonnier C Demchuk A Ziai W Anderson CS. Intracerebral haemorrhage: current approaches to acute management. Lancet. (2018) 392:1257–68. 10.1016/S0140-6736(18)31878-6 30319113

[22] Dong N Wang Y. MiR-30a Regulates S100A12-induced retinal microglial activation and inflammation by targeting NLRP3. Curr Eye Res. (2019) 44:1236–43. 10.1080/02713683.2019.1632350 31199706

[23] Hamzei Taj S Kho W Riou A Wiedermann D Hoehn M. MiRNA-124 induces neuroprotection and functional improvement after focal cerebral ischemia. Biomaterials. (2016) 91:151–65. 10.1016/j.biomaterials.2016.03.025 27031810

[24] Wang Z Lu G Sze J Liu Y Lin S Yao H. Plasma miR-124 is a promising candidate biomarker for human intracerebral hemorrhage stroke. Mol Neurobiol. (2018) 55:5879–88. 10.1007/s12035-017-0808-8 29101647 PMC5994210

[25] He XW Shi YH Liu YS Li GF Zhao R Hu Y. Increased plasma levels of miR-124-3p, miR-125b-5p and miR-192-5p are associated with outcomes in acute ischaemic stroke patients receiving thrombolysis. Atherosclerosis. (2019) 289:36–43. 10.1016/j.atherosclerosis.2019.08.002 31450012

[26] Deng S Jin P Sherchan P Liu S Cui Y Huang L. Recombinant CCL17-dependent CCR4 activation alleviates neuroinflammation and neuronal apoptosis through the PI3K/AKT/Foxo1 signaling pathway after ICH in mice. J Neuroinflammation. (2021) 18:62. 10.1186/s12974-021-02112-3 33648537 PMC7923481

[27] Greenberg LB Mofson R Fink M. Prospective electroconvulsive therapy in a delusional depressed patient with a frontal meningioma. A case report. Br J Psychiatry. (1988) 153:105–7. 10.1192/bjp.153.1.105 3224230

[28] Zhou L Wang D Qiu X Zhang W Gong Z Wang Y. DHZCP modulates microglial M1/M2 polarization via the p38 and TLR4/NF-kappaB signaling pathways in LPS-stimulated microglial cells. Front Pharmacol. (2020) 11:1126. 10.3389/fphar.2020.01126 32848745 PMC7406685

[29] Cui SY Zhang W Cui ZM Yi H Xu DW Liu W. Knockdown of long non-coding RNA LEF1-AS1 attenuates apoptosis and inflammatory injury of microglia cells following spinal cord injury. J Orthop Surg Res. (2021) 16:6. 10.1186/s13018-020-02041-6 33407665 PMC7786481

[30] Zhang Y Liu HL An LJ Li L Wei M Ge DJ. miR-124-3p attenuates neuropathic pain induced by chronic sciatic nerve injury in rats via targeting EZH2. J Cell Biochem. (2019) 120:5747–55. 10.1002/jcb.27861 30390343

[31] Liang YP Liu Q Xu GH Zhang J Chen Y Hua FZ. The lncRNA ROR/miR-124-3p/TRAF6 axis regulated the ischaemia reperfusion injury-induced inflammatory response in human cardiac myocytes. J Bioenerg Biomembr. (2019) 51:381–92. 10.1007/s10863-019-09812-9 31768721

[32] Dou Y Tian X Zhang J Wang Z Chen G. Roles of TRAF6 in central nervous system. Curr Neuropharmacol. (2018) 16:1306–13. 10.2174/1570159X16666180412094655 29651950 PMC6251041

[33] Qu X Wang N Cheng W Xue Y Chen W Qi M. MicroRNA-146a protects against intracerebral hemorrhage by inhibiting inflammation and oxidative stress. Exp Ther Med. (2019) 18:3920–8. 10.3892/etm.2019.8060 31656540 PMC6812313

[34] Yang Y Tan X Xu J Wang T Liang T Xu X. Luteolin alleviates neuroinflammation via downregulating the TLR4/TRAF6/NF-kappaB pathway after intracerebral hemorrhage. Biomed Pharmacother. (2020) 126:110044. 10.1016/j.biopha.2020.110044 32114357

[35] Ren H Kong Y Liu Z Zang D Yang X Wood K. Selective NLRP3 (Pyrin Domain-Containing Protein 3) inflammasome inhibitor reduces brain injury after intracerebral hemorrhage. Stroke. (2018) 49:184–92. 10.1161/STROKEAHA.117.018904 29212744 PMC5753818

[36] Ma Q Chen S Hu Q Feng H Zhang JH Tang J. NLRP3 inflammasome contributes to inflammation after intracerebral hemorrhage. Ann Neurol. (2014) 75:209–19. 10.1002/ana.24070 24273204 PMC4386653

[37] Song HL Zhang SB. Therapeutic effect of dexmedetomidine on intracerebral hemorrhage via regulating NLRP3. Eur Rev Med Pharmacol Sci. (2019) 23:2612–9. 10.26355/eurrev_201903_17411 30964190

